# Integrin β3 and LKB1 are independently involved in the inhibition of proliferation by lovastatin in human intrahepatic cholangiocarcinoma

**DOI:** 10.18632/oncotarget.6238

**Published:** 2015-10-26

**Authors:** Sheng-Huei Yang, Hung-Yun Lin, Chun A Changou, Chun-Han Chen, Yun-Ru Liu, Jinghan Wang, Xiaoqing Jiang, Frank Luh, Yun Yen

**Affiliations:** ^1^ PhD Program for Cancer Biology and Drug Discovery, College of Medical Science and Technology, Taipei Medical University, Taipei, Taiwan; ^2^ Taipei Cancer Center, Taipei Medical University, Taipei, Taiwan; ^3^ Integrated Laboratory, Center of Translational Medicine, Taipei Medical University, Taipei, Taiwan; ^4^ Core Facility, Taipei Medical University, Taipei, Taiwan; ^5^ Office of Human Research, Taipei Medical University, Taipei, Taiwan; ^6^ The First Department of Biliary Surgery, Eastern Hepatobiliary Surgical Hospital, Second Military Medical University, Shanghai, China; ^7^ School of medicine, Taipei Medical University, Taipei, Taiwan; ^8^ Sino-American Cancer Foundation, Arcadia, California, United States

**Keywords:** Bile duct cancer, HMG-CoA reductase inhibitor, integrin, LKB1, TGF-β1

## Abstract

Human intrahepatic cholangiocarcinomas are one of the most difficult cancers to treat. In our study, Lovastatin, a 3-hydroxy-3-methylglutaryl-coenzyme-CoA (HMG-CoA) reductase inhibitor, demonstrated anticancer properties by inhibiting cancer cell proliferation, cell migration and cell adhesion. Lovastatin inhibited the expressions of transforming growth factor (TGF)-β1, cyclooxygenase (COX)-2, and intercellular adhesion molecule (ICAM)-1. Furthermore, lovastatin inhibited the expressions of integrin β1 and integrin β3 but not integrin αv or integrin β5. While Lovastatin's inhibitory effects on TGFβ1, COX2, and ICAM-1 expression were independently controlled by the tumor suppressor LKB1, integrin β3 expression was not affected. Lovastatin's inhibitory effect on cell adhesion was associated with the decreased expression of integrin β3 and cell surface heterodimer integrin αvβ3. Quantitative real time PCR, fluorescent microscopy, and cell migration assays all confirmed that Lovastatin inhibits integrin αvβ3 downstream signaling including FAK activation, and β-catenin, vimentin, ZO-1, and β-actin. Overall, Lovastatin reduced tumor cell proliferation and migration by modifying the expression of genes involved in cell adhesion and other critical cellular processes. Our study highlights novel anti-cancer properties of Lovastatin and supports further exploration of statins in the context of cholangiocarcinoma therapy.

## INTRODUCTION

Cholangiocarcinomas are malignant tumors of the biliary tract and are the second most common type of primary liver cancer. Biliary tract cancer is associated with a high mortality rate due to its difficulty of early detection and resistance to most chemotherapeutic agents. Several integrins expressed in cholangiocarcinomas [1–3] have been reported in studies related to cancer pathogenesis. Those cell surface integrins are known to regulate cell proliferation [4], migration [5], invasion [1, 5], angiogenesis [6], and adhesion [7].

3-Hydroxy-3-methylglutaryl (HMG)-CoA reductase inhibitors, also called statins, are commonly used as lipid-lowering drugs that inhibit cholesterol biosynthesis [8]. Statins inhibit the production of isoprenoids and thus block the cholesterol biosynthetic pathway [9]. They are used therapeutically to upregulate low-density lipoprotein (LDL) receptor-mediated removal of plasma cholesterol in the liver. In addition to their lipid-lowering properties, statins have been linked to a variety of physiological processes. Lovastatin can delay the onset and progression of diabetic nephropathies in part, through suppressing the glomerular expression of TGF-β1, independent of its cholesterol-lowering effect [10]. Simvastatin can influence alveolar bone remodeling by regulating the expression of growth factors crucial to osteogenesis [11]. It also inhibits transforming growth factor (TGF)-β1-induced fibronectin expression in asthmatic fibroblasts [12]. In the context of cancer, simvastatin mediates suppress prostate cancer PC3 micrometastasis through inhibition of integrin αvβ3 activity and suppression of the interaction between prostate cancer cell integrin αvβ3 and endothelial intercellular adhesion molecule (ICAM)-1 [13]. Simvastatin has also been found to reduce tumor cell adhesion in human peritoneal mesothelial cells by decreasing expressions of VCAM-1 and β1 integrin [14]. Studies on lovastatin suggest that it can also induce cytoskeletal alterations and ultimately modulate adhesion, motility, and proteolysis [15].

Recent studies have started to explore the possibility of using statins to treat bile duct cancers [16–22]. In fact, clinical studies have looked at bile duct cancer in simvistatin monotherapy [23] or combination therapy with anti-cancer drug, S-1 [16]. Simvastatin stimulates anti-proliferation in cholangiocarcinomas by inhibiting Rac1 activity [23] or downregulating E2F-1/TS [16, 23]. Similar studies have reported about lovastatin in inhibiting cell proliferation by depleting geranylgeranyl pyrophosphate through Skp2 pathway degradation [24]. Lovastatin also shows to overcome gefitinib-resistance cholangiocarcinomas [25], and enhance adenovirus-mediated TRAIL induced apoptosis in prostate cancer [26]. Overall, statins seem to reduce the risk of cholangiocarcinomas [27]. However, this exact mechanism remains to be fully understood.

We hypothesize that lovastatin is able to inhibit proliferation, cell migration and cellular adhesion in cholangiocarcinomas through the integrin and TGF-β1-dependent pathways. In this study, two human intrahepatic cholangiocarcinoma cell lines, RBE and HuH-28, were treated with lovastatin and multiple studies including, qPCR, flow cytomemetry, cell migration assay, and trypan blue exclusion assay were conducted to demonstrate our theory on lovastatin's anti-proliferative properties on cellular mechanisms. Overall, this study confirmed the therapeutic potential of lovastatin against human intrahepatic cholangiocarcinoma and provided support for further investigation of statins in relation to cancer therapy and drug development.

## RESULTS

### Lovastatin regulates gene expressions in HuH-28 and RBE human cholangiocarcinoma cells

**Figure 1:**
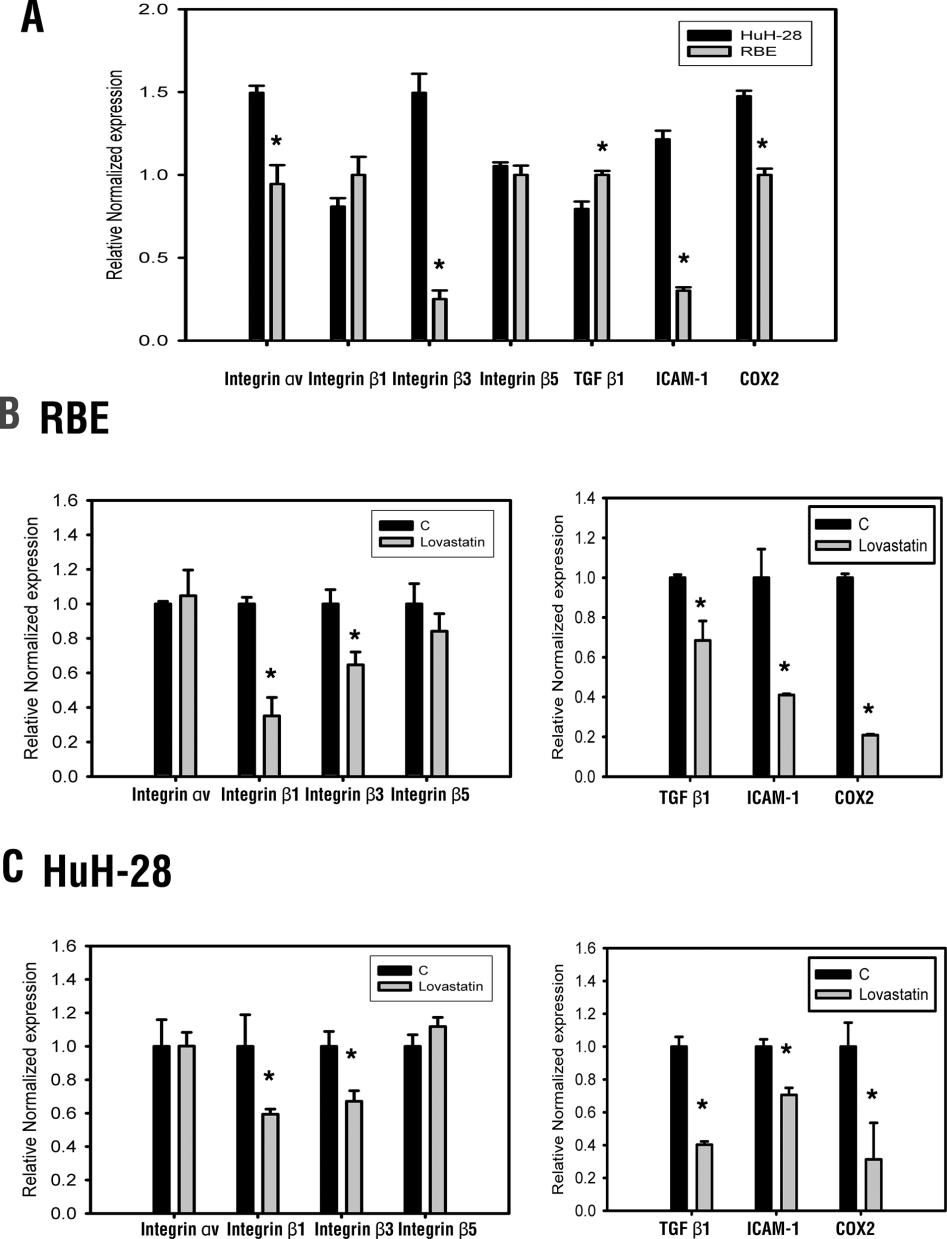
Lovastatin regulates gene expression (**A**) mRNA expressions of integrin αv, β1, β3, β5, TGFβ1, COX2, and ICAM-1 in RBE and HuH-28 cell lines. (**B**) RBE and (**C**) HuH-28 cells (10^6^ cells/well) were treated with lovastatin (L) for 24 h. Cells were harvested, and total RNA was extracted. mRNA expressions of integrin αv, β1, β3, β5, TGFβ1, COX2, and ICAM-1 were detected using a qPCR, as described in “Materials and Methods”. Student's *t* test was conducted and considered significant at *p* < 0.05 (*).

**Figure 2:**
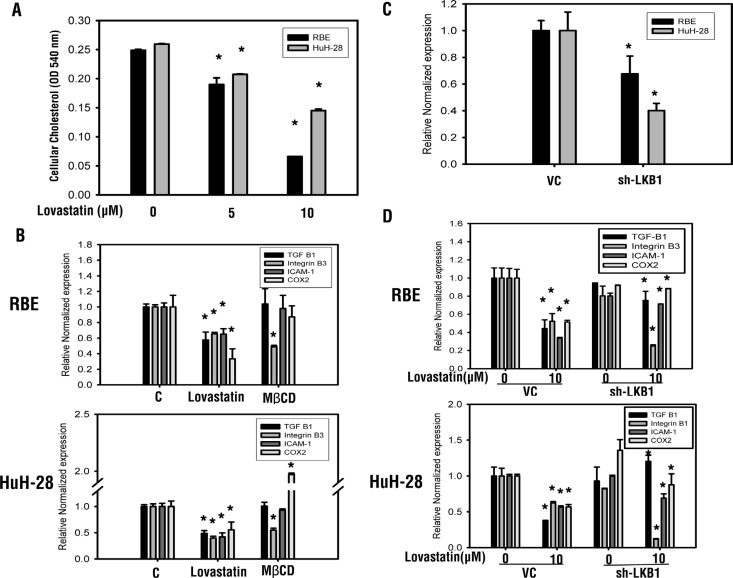
Lovastatin regulates gene expressions through cholesterol depletion or LKB1 activity (**A**) RBE and HuH-28 cells (10^8^ cells/dish) were treated with lovastatin for 24 h, 10^6^ cells were counted, and cholesterol was purified and detected with a cholesterol assay kit (Biovision). (**B**) RBE and HuH-28 cells (10^6^ cells/well) were treated with lovastatin (L) for 24 h or 10 μM MβCD for 6 h. Cells were harvested, and total RNA was extracted. mRNA expressions of integrin αv, β1, β3, β5, TGFβ1, COX2, and ICAM-1 were detected using a qPCR. (**C**) RBE and HuH-28 cells were stably transfected with an shLKB1 plasmid and selected by puromycin. Cells (10^6^ cells/well) were treated with lovastatin (L) for 24 h, cells were harvested, and total RNA was extracted. LKB1 mRNA expression was detected by a qPCR. (**D**) mRNA expressions of integrin *αv*, β1, β3, β5, TGFβ1, COX2, and ICAM-1 were detected using a qPCR, as described in “Materials and Methods”. Student's *t* test was conducted and considered significant at *p* < 0.05 (*).

In order to investigate the anti-proliferation mechanisms associated with lovastatin, several diagnostic markers of cholangiocarcinomas were studied in cells treated with lovastatin (Figure 1A). As shown in Figure 1B and 1C, lovastatin inhibited the expression of integrin *β1* and integrin *β3*, but not integrin *αv* or integrin *β5*. At the same time, the expression of *TGF-β1*, *COX-2,* and *ICAM-1* were also inhibited by lovastatin (Figure 2). These results indicate that lovastatin changes the expression of several genes in cholangiocarcinomas through multiple pathways.

### Multiple mechanisms are involved in lovastatin-induced anti-proliferation in cholangiocarcinomas

Known for its ability to reduce cholesterol synthesis, lovastatin was tested for its ability to decrease cholesterol levels in human intrahepatic cholangiocarcinoma cell lines. RBE and HuH-28 cells (10^8^ cells/dish) were treated with lovastatin for 24 h. MβCD was used as a positive control of cholesterol depletion. A decrease in cholesterol levels correlated with increased lovastatin concentrations in a dose dependent manner (Figure 2A). Compared to HuH-28, RBE cells were more sensitive to lovastatin in terms of cellular cholesterol levels. In order to elucidate the potential role of lovastatin in cancer gene expression, lovastatin-treated HuH-28 and RBE cells were harvested and qPCR was used to quantify expression of *integrin β3*, *TGFβ1, ICAM-1*, and *COX-2*. Results in Figure 2B indicate that both lovastatin and MβCD reduced the expression of integrin β3. But unlike MβCD, only lovastatin decreased the expressions of TGFβ1, COX2, and ICAM-1. Lovastatin was reported to induce LKB1 activation. After knockdown of LKB1 gene (Figure 2C), we found decreased expression of *TGFβ1, COX2,* and *ICAM-1* (Figure 2D). In contrast, inhibition of integrin β3 mRNA accumulation by lovastatin was enhanced (Figure 2D). These results suggest that lovastatin affects expression of genes via different mechanisms.

### Lovastatin inhibits the integrin/β-catenin pathway and decreases β-actin in cholangiocarcinomas

**Figure 3:**
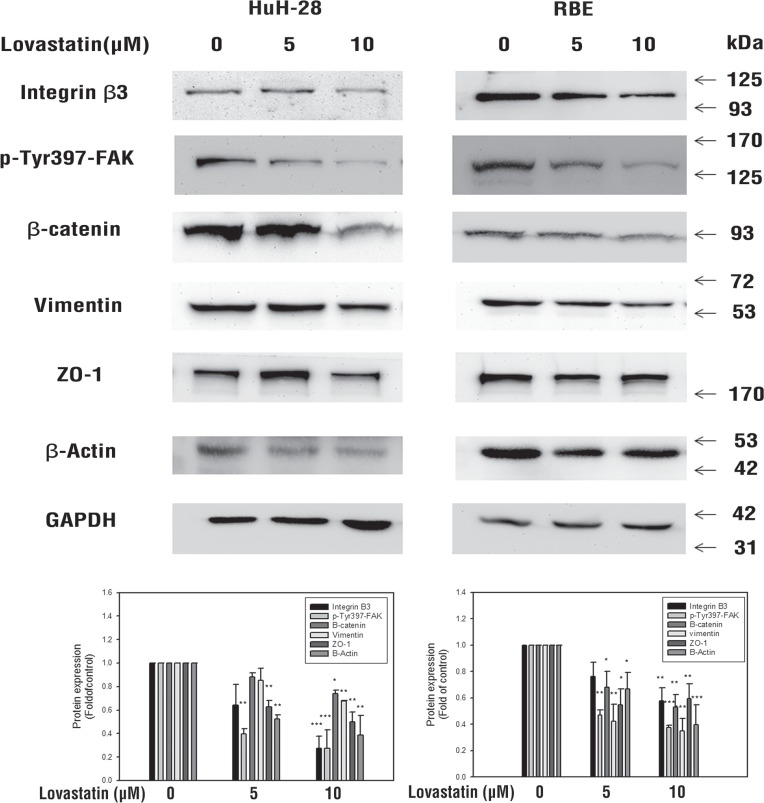
Lovastatin inhibits integrinβ3/β-catenin pathway protein expression Cells (10^6^ cells/well) were seeded in a 6-well tray and treated with lovastatin for 24 h. Cells were harvested, and total proteins were extracted. The integrin β3 and β-catenin pathway, including integrin β3, p-FAK, vimentin, ZO-1, and β-actin, were detected using Western blotting analyses. Student's *t* test was conducted and considered significant at *p* < 0.05 (*); *p* < 0.01(**); *p* < 0.005(***).

**Figure 4:**
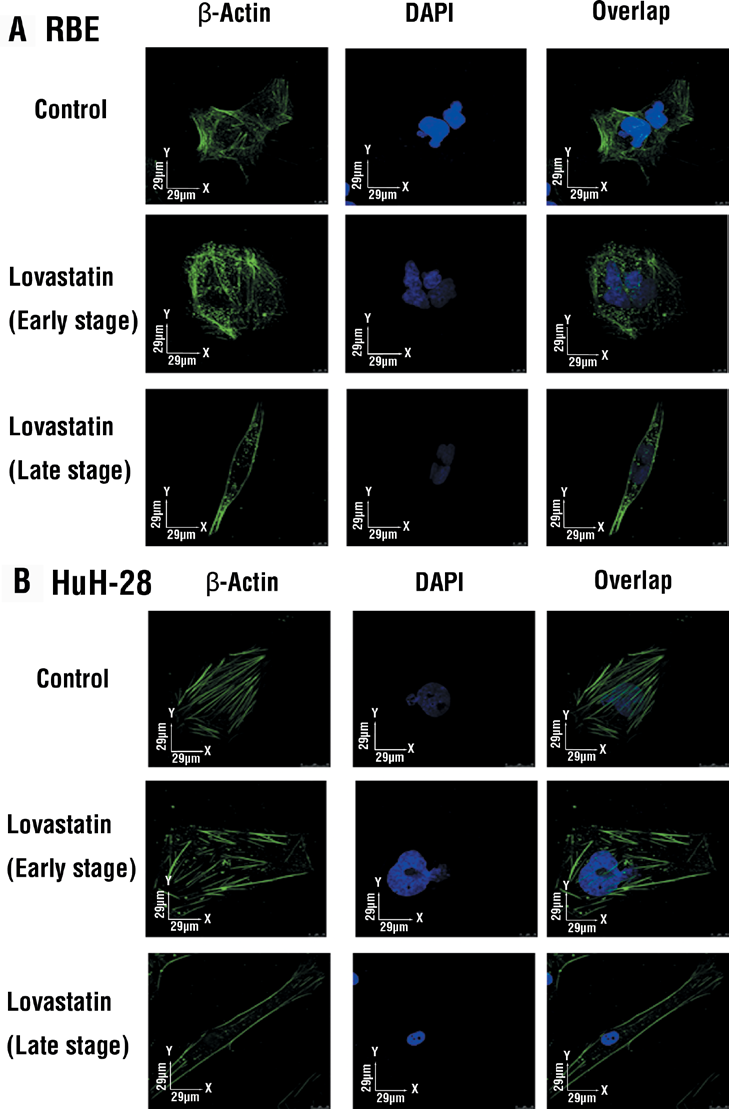
Lovastatin disrupts β-actin filament formation Cells (10^3^ cells/well) were seeded in the chamber side, and treated with lovastatin for 24 h. Cells were fixed and stained with a β-actin antibody and DAPI, and β-actin filaments were detected by a confocal microscopic assay.

Lovastatin inhibited the mRNA expression of integrin β3 (Figure 1B, 1C) and protein accumulation (Figure 3). It was interesting to note Lovastatin's downstream signaling effects. Results in Figure 3 indicate that lovastatin suppressed the accumulation of integrin β3 and decreased signaling molecules downstream to integrin β3, including p-FAK, vimentin, ZO-1, and β-catenin. Decreased β-actin-induced filament fragments were also observed in cells treated with lovastatin (Figure 4). These results suggest that lovastatin inhibits cellular migration and actin filamentation by inhibiting integrin β3 expression and function.

### Lovastatin inhibits cancer cell adhesion by inhibiting presentation of the cell surface integrin, αvβ3

**Figure 5:**
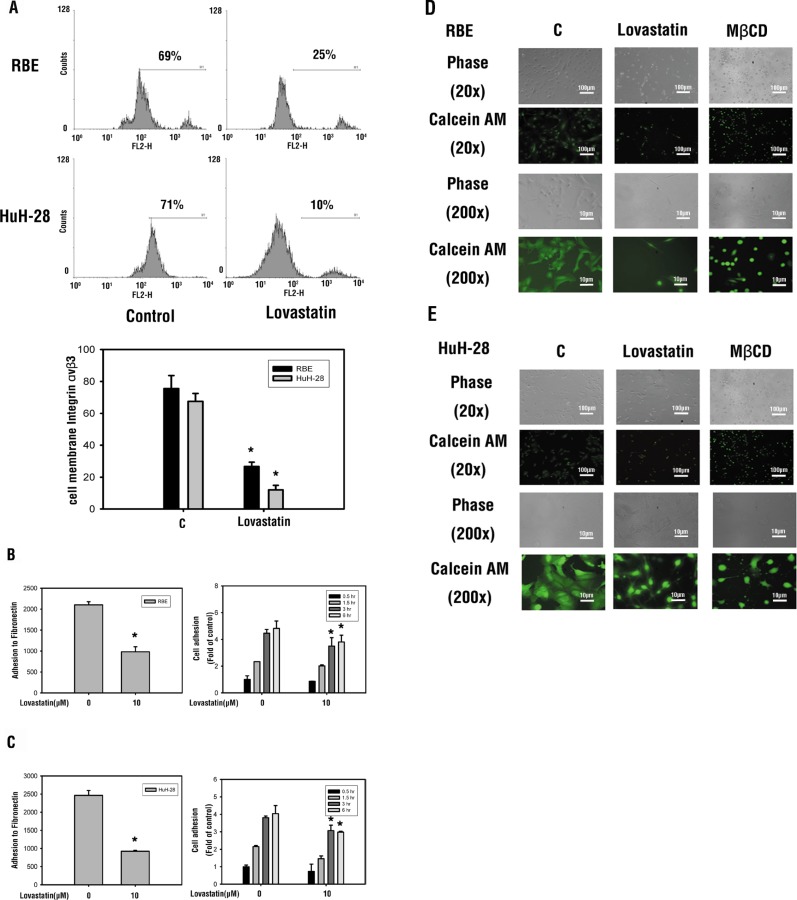
Lovastatin deceases cell surface integrin αvβ3 presentation and downregulates the adhesion ability (**A**) RBE and HuH-28 cells (10^6^ cells/well) were treated with lovastatin for 24 h. Cells were harvested, and cell surface integrin αvβ3 was detected by flow cytometry. Student's *t*-test was conducted, and results were considered significant at *p* < 0.05 (*) (**B**) RBE and (**C**) HuH-28 cells (10^4^ cells/well) were starved in 0.1% serum-containing medium with 10 μM lovastatin at 37°C for 4 h, seeded into a plate coated with fibronectin for 30 min, and seeded into a 24-well cell culture plate for 0.5, 1.5, 3, and 6 h. The attached cells were quantified using a fluorometric detection system (Millipore). Data are expressed as the mean ± S.D. of triplicate determinations. (**D**) RBE and (**E**) HuH-28 cell lines (10^6^ cells/well) were seeded in a 6-well tray, treated with lovastatin for 24 h, and assessed using calcein AM. Calcein AM is a fluorogenic esterase substrate that is hydrolyzed to a green-fluorescent product (casein) only in living cells. Student's *t* test was conducted and considered significant at *p* < 0.05 (*).

To confirm whether lovastatin-induced integrin expression is involved in anti-proliferation, we studied heterodimers of the cell surface integrin (αvβ3) through flow cytometry. As shown in Figure 5A, heterodimers of the cell surface integrin αvβ3 were downregulated by lovastatin exposure in both cell lines. Moreover, lovastatin exposure to HuH-28 and RBE cell lines negatively impacted the ability of αvβ3 to adhere to fibronectin (Figure 5B). Overall, as the exposure to lovastatin increased, cellular adhesion properties of HuH-28 and RBE cells decreased (Figure 5C). Studies of fluorescence microscopy confirmed the morphological cellular changes induced by lovastatin (Figure 5D). These results suggest that lovastatin reduced the expression of *integrin β3* and its presentation on the cell surface, thus affecting growth, adhesion, morphology, and migration of cancer cells.

### Lovastatin inhibits cell proliferation and cell migration in RBE and HuH-28 cholangiocarcinoma cells

**Figure 6:**
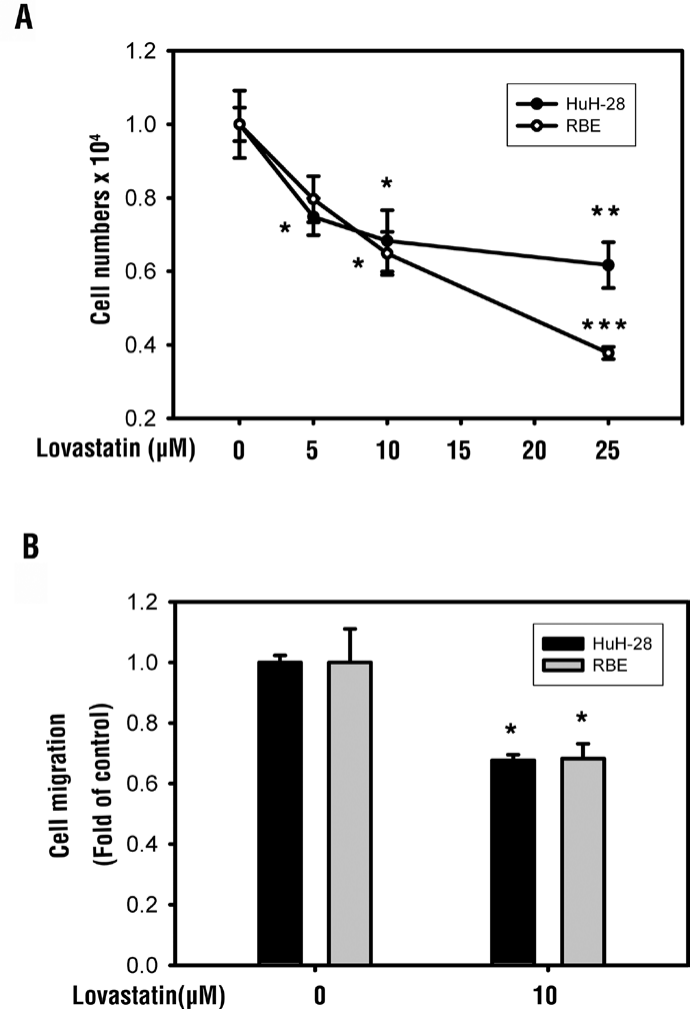
Lovastatin inhibits cell proliferation and cell migration in RBE and HuH-28 cholangiocarcinoma cells (**A**) RBE and HuH-28 cells (10^3^ cells/well) were treated with lovastatin (L) for 72 h. Cell numbers were detected using a trypan blue assay. (**B**) RBE and HuH-28 cells (10^5^ cells/well) were starved in 0.1% serum-containing medium with 10 μM lovastatin at 37°C for 4 h, and seeded into the upper chamber of a transwell using the Millipore system for cell migration. Cells were chemoattracted for 24 h to migrate through a membrane and were quantified using a fluorometric detection system (Millipore). Data are expressed as the mean ± S.D. of triplicate determinations. Student's *t* test was conducted and considered significant at *p* < 0.05 (*); *p* < 0.01(**); *p* < 0.005(***).

In order to examine the antiproliferative effect of lovastatin, RBE and HuH-28 cells (10^3^ cells/well), were treated with lovastatin for 72 h. The antiproliferative effect was quantified by calculating cell number. Trypan blue exclusion was used. Our data confirmed that lovastatin inhibited cell proliferation in both bile duct cancer cell lines (Figure 6A). The effect of lovastatin on cell migration was also studied. RBE and HuH-28 cells (10^5^ cells/well) were seeded in the upper chamber of a transwell using the Millipore system for cell migration. Lovastatin reduced cell migration in both cancer cell lines (Figure 6B). Ultimately, these results suggest that lovastatin has ability to reduce cell proliferation and migration in cholangiocarcinoma cell lines.

These results also suggest that the lovastatin activates two signal transduction pathways to induce anticancer progression. One pathway involves the expression of integrin β3/β1, which affects FAK activity, downstream gene expression and signal transduction. The other pathway is via LKB1 activation, which inhibits TGF-β activity and downstream biological activities. Cross-talk between these two pathways likely induces anti-proliferation and inhibits cancer migration and metastasis.

## DISCUSSION

Lovastatin inhibited proliferation of RBE and HuH-28 human intrahepatic cholangiocarcinoma cells (Figure 6A). Lovastatin exposure decreased cellular attachment (Figure 5C) and induced changes in cell morphology and integrity (Figure 5D). Because exposure to MβCD and lovastatin induced similar results (Figure 5D), cholesterol depletion by MβCD causes anoikis-like apoptosis, which in A431 cells involved decreased raft levels, Bcl-xL downregulation, caspase-3 activation, and Akt inactivation regardless of epidermal growth factor receptor activation [28]. Pretreatment with cholesterol alone stimulated an increase in the number of viable cells in Mz-ChA-1 cholangiocarcinoma cells, and fully restored cell viability following simvastatin treatment [23]. These studies suggest that the exact mechanism of lovastatin-induced anti-proliferation may involve cholesterol depletion.

While exposure to lovastatin and MβCD both reduced the expression of *integrin β3* (Figure 2B), only lovastatin decreased the expressions of *TGFβ1, COX2,* and *ICAM-1* (Figure 2B), suggesting that cholesterol depletion may not be the only mechanism involved in lovastatin-induced anti-proliferation. LKB1-phosphorylation of Smad4 at Thr-77 of its DNA-binding domain inhibits Smad4 from binding to TGF-β-specific promoter sequences; this correlates with the negative regulatory effect that LKB1 exerts on Smad4-dependent transcription [29]. Lovastatin is reported to induce LKB1 activation [25, 30]. Both ICAM-1 and COX2 are stimulated by TGF-β1 [31, 32]. After knockdown of LKB1 gene expression, lovastatin-induced inhibitory effects on the expressions of TGFβ1, COX2, and ICAM-1 were reduced (Figure 2D). These results indicate that lovastatin suppresses the expressions of *TGFβ1, COX2,* and *ICAM-1* through LKB1 activation.

Cell migration and cell adhesion abilities were also blocked by lovastatin (Figures 5B, 5C, 6B). Lovastatin downregulated the integrin β3/FAK pathway (Figure 3). It also reduced cell surface integrin αvβ3 expression (Figure 5B) and the binding ability to fibronectin (Figure 5C). Fibronectin regulates cell growth in cholangiocarcinomas [33]. Fibronectin is known to induce activation of FAK via extracellular signal-regulated kinase (ERK) or PI3K/Akt to increase matrix metalloproteinase (MMP)-9/calpain-2 or MMP-9/RhoA activity, respectively, and lead to lung cancer metastasis [34]. Those studies suggested that lovastatin is able to inhibit cancer migration and metastasis by downregulating either the FAK-ERK1/2 or FAK-PI3K pathway.

Statins reduce the isoprenoids, farnesyl and geranylgeranyl pyrophosphate, which are essential intermediates in cellular events and properties, including cytoskeletal integrity, adhesion, migration, and viability [35]. Simvastatin disrupts the actin cytoskeleton and focal adhesion complexes by a cholesterol synthesis-independent mechanism [35]. However, results indicated that lovastatin inhibited integrin β3 by cholesterol depletion in present studies (Figure 2). Furthermore, β-catenin, a downstream signal of integrin β3, was simultaneously reduced in lovastatin treatment. Moreover, when β-catenin decreases, related proteins such as FAK, VE-cadherin, vinculin, and β-actin simultaneously decreases [36]. We postulate that lovastatin inhibits actin filaments through inhibiting integrin β3.

In conclusion, several mechanisms proposed from present studies are involved in lovastatin-inhibited inhibitory effects on proliferation, migration, and adhesion in cholangiocarincoma. Treatment of human intrahepatic cholangiocarcinomas with lovastatin induced cholesterol depletion and LKB1 activation (Figure 2). The cholesterol-depleting effect of lovastatin inhibited the expression of *integrin β3* and cell surface heterodimer integrin αvβ3 presentation. Additionally, the integrin αvβ3 downstream signaling including FAK activation and organization of β-catenin, vimentin, ZO-1 and β-actin were inhibited by cholesterol depletion (Figure 3). Activation of LKB1 by lovastatin blocked TGF-β expression and activation, which controls functions of COX2 and ICAM-1 (Figure 2D).

**Figure 7:**
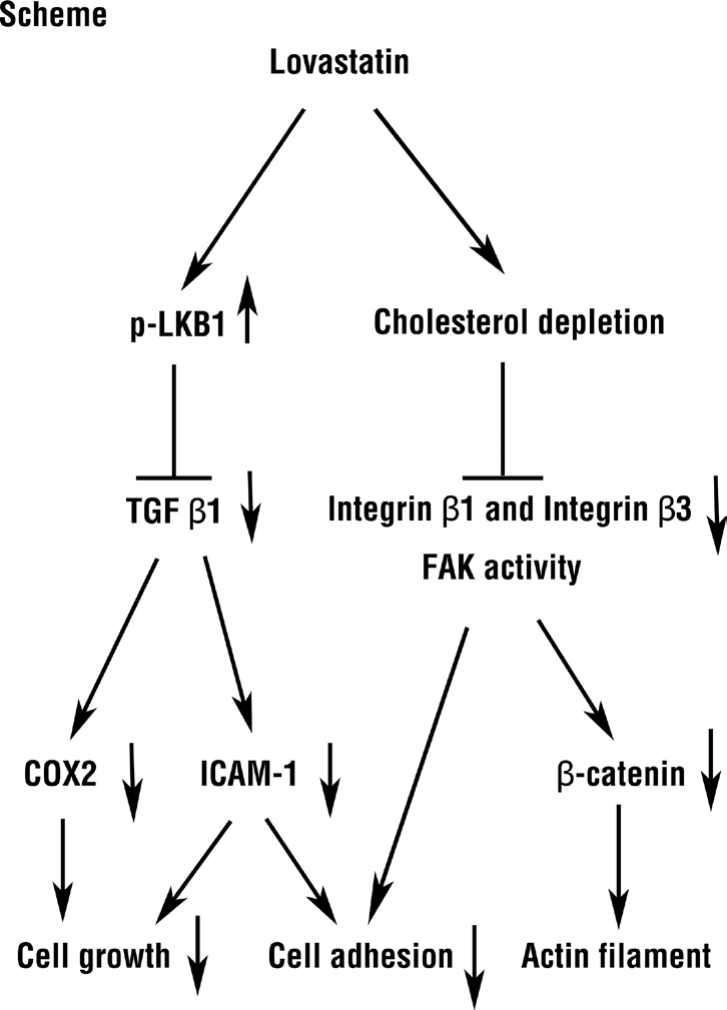
Roles of lovastatin regulated mechanism in RBE and HuH-28 human intrahepatic cholangiocarcinoma cells Lovastatin inhibited cell proliferation and cell adhesion through TGFβ1 to block COX2 and ICAM-1 mRNA expression by LKB1, and downregulated integrin β3/FAK pathway by inhibited integrin β3 expression.

Clinically, patients treated with statins have been associated with a reduced risk of cholangiocarcinomas [27]. However, this regulatory mechanism is poorly understood. Here, we propose a novel pathway in which lovastatin inhibits human intrahepatic cholangiocarcinoma proliferation (Figure 7) and strongly encourage further studies to explore statins potential therapeutic role in intrahepatic cholangiocarcinoma. As an approved agent, lovastatin may one day impact therapy and should be considered in future clinical trials related to cancers, particularly intrahepatic cholangiocarcinoma.

## MATERIALS AND METHODS

### Cell lines

Two human cholangiocarcinoma cell lines (RBE and HuH-28) were purchased from RIKEN Bioresource Center (Ibaraki, Japan) and maintained for the study in RPMI-1640 or minimum essential medium (MEM) containing 10% fetal bovine serum (FBS) and P/S solution (Invitrogen) in a 5% CO_2_ incubator at 37°C.

### Short hairpin (sh)RNA transfection

Human cholangiocarcinoma cells were seeded into six-well tissue culture plates until 60%~80% confluence and maintained in the absence of antibiotics for 24 h before transfection. The culture medium was removed before transfection, and cells were washed once with phosphate-buffered saline (PBS), then transfected with short hairpin (sh)-RNA for LKB1: CATCTACACTCAGGACTTCAC shRNA or scrambled RNA (2 μg/well) purchased from the National RNAi Core Facility (Academia Sinica, Taipei, Taiwan) using lipofectamine 2000 (2 μg/well) in Opti-MEM I medium according to the manufacturer's instructions (Invitrogen). After transfection, cultures were incubated at 37°C for 4 h, and then placed in fresh culture medium. After an additional 72 h, cells were selected by puromycin. Selected cells were used in the experiments.

### Quantitative real-time polymerase chain reaction (qPCR)

Total RNA was extracted using an RNeasy Micro Kit (Qiagen, Venlo, the Netherlands), and complementary cDNA synthesis was performed using the RevertAid^™^ H Minus First Strand cDNA Synthesis Kit (Thermo Scientific, Rockford, IL). A qPCR was conducted with 5 μL of DNA combined with 10 μL of IQ SYBR Green supermix (Bio-Rad, Hercules, CA), 0.3 μL each of 20 μM of forward and reverse primers, and 4.7 μL DNase/RNase-free water. Sequences for the amplified primers are listed as follows: integrin αv forward 5′-TCCGATTCCAAACTGGGAGC-3′ and reverse 5′-AAGGCCACTGAAGATGGAGC-3′, TGFβ1 forward 5′-TGGTGGAAACCCACAACGAA-3′ and reverse 5′-GAGCAACACGGGTTCAGGTA-3′; integrin β3 forward 5′-CGAGTGCCTCTGTGGTCAAT-3′ and reverse 5′-AGAAGTCGTCACACTCGCAG-3′; integrinβ1 forward 5′-CCTACTTCTGCACGATGTGATG-3′ and reverse 5′-CCTTTGCTACGGTTGGTTACATT-3′; integrin β5 forward 5′-AACTCGCGGAGGAGATGAG-3′ and reverse 5′- GGTGCCGTGTAGGAGAAAGG-3′; ICAM-1 forward 5′-TATGGCAACGACTCCTTCT-3′ and reverse 5′-CATTCAGCGTCACCTTGG-3′; COX2 forward 5′-GCCAAGCACTTTTGGTGGAG-3′ and reverse 5′-GGGACAGCCCTTCACGTTAT-3′; and 18s forward 5′-GTAACCCGTTGAACCCCATT-3′ and reverse 5′-CCATCCAATCGGTAGTAGCG-3′. Reactions were performed in a CFX Connect^™^ Real-Time PCR Detection System (Bio-Rad).

### Transwell cell migration assay

A cell migration assay was conducted with a transwell system. Briefly, cells were trypsinized and adjusted to 10^5^ cells/ml in a cell suspension. Cells in a 200-μl volume was seeded into the upper chamber of a transwell, and 800 μl of medium with 10% stripped FBS was added to the lower chamber. Cells were then cultured at 37°C for 6 h, and cells were chemoattracted to pass through the membranes and were quantified using a fluorometric detection system (Millipore). The fluorometric signals were determined by an Epoch enzyme-linked immunosorbent assay (ELISA) reader (Biotek) at 485/538 nm.

### Cell adhesion assay

Cells were trypsinized, washed twice with PBS, and resuspended in serum-free medium containing lovastatin at 10^6^ cells/ml. After incubation at 37°C for 30 min, cells were washed twice with warm medium and resuspended in serum-free medium at 10^6^ cells/ml, and 200 μl of the cell suspension was added to 24-well plates coated with fibronectin. Cells were incubated at 37°C for 0.5, 1.5, and 3 h. Adherent cells were counted with a CyQUANT Cell Proliferation Assay Kit (Life Technologies), and the fluorescence was recorded using a plate reader (Plate CHAMELEON, HIDEX, Turku, Finland) with a fluorescence filter set (excitation at 485 nm and emission at 530 nm).

### Flow cytometric assay

Cells were harvested with trypsin-EDTA and resuspended in cold PBA (1.0% bovine serum albumin (BSA) and 0.1% sodium azide in PBS), followed by incubation with an anti-integrin αvβ3 antibody (Millipore, Temecula, CA) at 4°C for 60 min. After washing in PBA, cells were resuspended with Alexa Fluor-conjugated second antibodies (Life Technologies, NY); 1:50 in PBA). After an additional 60 min at 4°C, cells were washed and analyzed by flow cytometry (FACSCalibur flow cytometer, Becton Dickinson, NJ), and 10^4^ events were collected and analyzed using WinMDI 2.9 software.

### Immunoblotting

After treatment, cells were lysed in cell lysis buffer (10 mM Tris at pH 7.4, 150 mM NaCl, 0.2% Triton X-100, 2 mM EDTA, 1 mM PMSF, and 1 × protease inhibitor mixture), and the protein concentration was determined using a BCA assay (Thermo Scientific, Rockford, IL). Cell lysates were separated on sodium dodecylsulfate polyacrylamide gel electrophoresis (SDS-PAGE) and transferred to nitrocellulose membranes; the membranes were probed with antibodies against ZO-1, vimentin, and GAPDH purchased from Genetex (San Antonio, TX), and p-Tyr397-FAK purchased from Cell Signaling Technology (Beverly, MA). β-Catenin and β-actin were purchased from BD Biosciences (San Jose, CA). Integrin β3 was purchased from Santa Cruz (Santa Cruz, CA). Secondary antibodies were either goat anti-rabbit immunoglobulin G (IgG) or rabbit anti-mouse IgG (1:3000), depending on the origin of the primary antibody. Immunoreactive proteins were detected using the BioSpectrum 810 Imaging System (UVP).

### Fluorescence confocal microscopy

Cells grown on chamber slides were treated with lovastatin for 24 h at 37°C. Cells were fixed in 4% paraformaldehyde for 10 min at room temperature. Slides were washed with PBS and incubated with 5% BSA (Sigma) in PBS containing 0.05% Tween-20 for 1 h to block nonspecific binding sites and for permeabilization. Anti-β-actin antibodies were applied, and slides were incubated overnight at 4°C. After three rinses with PBS, slides were incubated with a mixture of Alexa Fluor^®^ 488 goat anti-mouse IgG (Invitrogen, 1:150 in TBS containing 3% BSA) for 30 min. Slides were mounted with an anti-fade, water-based mounting medium with 4,6-diamidino-2-phenylindole (DAPI; Sigma) and analyzed under a laser scanning confocal fluorescence microscope (Leica TCS SP5, Heidelberg, Germany). Excitation wavelengths of 365 (for DAPI) and 488 nm were respectively used to generate fluorescence emissions in blue and green.

### Transwell cell migration assay

A cell migration assay was conducted with a transwell system. Briefly, cells were trypsinized and adjusted to 10^5^ cells/ml of a cell suspension. Cells in a 200-μl volume were seeded into the upper chamber of a transwell, and 800 μl of medium was added with 10% stripped FBS in the lower chamber. Cells were then cultured at 37°C for 6 h, were chemoattracted to pass through the membranes, and were quantified using a fluorometric detection system (Millipore). The fluorometric signals were determined with a plate reader (Plate CHAMELEON, HIDEX) at 485/538 nm.

### Data analysis and statistics

Immunoblot and nucleotide densities were measured with a Storm 860 Phosphorimager, followed by analysis with ImageQuant software (Molecular Dynamics, Sunnyvale, CA). Student's *t*-test was conducted, and results were considered significant at *p* < 0.05 (*), *p* < 0.01 (**) and *p* < 0.005(***).
